# The Use of Automated Machine Translation to Translate Figurative Language in a Clinical Setting: Analysis of a Convenience Sample of Patients Drawn From a Randomized Controlled Trial

**DOI:** 10.2196/39556

**Published:** 2022-09-06

**Authors:** Hailee Tougas, Steven Chan, Tara Shahrvini, Alvaro Gonzalez, Ruth Chun Reyes, Michelle Burke Parish, Peter Yellowlees

**Affiliations:** 1 Department of Psychiatry and Behavioral Sciences University of California, Davis Sacramento, CA United States; 2 Department of Psychiatry and Behavioral Sciences Stanford University School of Medicine Stanford, CA United States

**Keywords:** telepsychiatry, automated machine translation, language barriers, psychiatry, assessment, automated translation, automated, translation, artificial intelligence, AI, speech recognition, limited English proficiency, LEP, asynchronous telepsychiatry, ATP, automated speech recognition, ASR, AMT, figurative language device, FLD, language concordant, language discordant, AI interpretation

## Abstract

**Background:**

Patients with limited English proficiency frequently receive substandard health care. Asynchronous telepsychiatry (ATP) has been established as a clinically valid method for psychiatric assessments. The addition of automated speech recognition (ASR) and automated machine translation (AMT) technologies to asynchronous telepsychiatry may be a viable artificial intelligence (AI)–language interpretation option.

**Objective:**

This project measures the frequency and accuracy of the translation of figurative language devices (FLDs) and patient word count per minute, in a subset of psychiatric interviews from a larger trial, as an approximation to patient speech complexity and quantity in clinical encounters that require interpretation.

**Methods:**

A total of 6 patients were selected from the original trial, where they had undergone 2 assessments, once by an English-speaking psychiatrist through a Spanish-speaking human interpreter and once in Spanish by a trained mental health interviewer-researcher with AI interpretation. 3 (50%) of the 6 selected patients were interviewed via videoconferencing because of the COVID-19 pandemic. Interview transcripts were created by automated speech recognition with manual corrections for transcriptional accuracy and assessment for *translational* accuracy of FLDs.

**Results:**

AI-interpreted interviews were found to have a significant increase in the use of FLDs and patient word count per minute. Both human and AI-interpreted FLDs were frequently translated inaccurately, however FLD translation may be more accurate on videoconferencing.

**Conclusions:**

AI interpretation is currently not sufficiently accurate for use in clinical settings. However, this study suggests that alternatives to human interpretation are needed to circumvent modifications to patients’ speech. While AI interpretation technologies are being further developed, using videoconferencing for human interpreting may be more accurate than in-person interpreting.

**Trial Registration:**

ClinicalTrials.gov NCT03538860; https://clinicaltrials.gov/ct2/show/NCT03538860

## Introduction

The most recent US Census Bureau investigation records that nearly 26 million individuals older than 5 years are considered of limited English proficiency (LEP), with a reduced ability to speak, write, or read English [1]. In the United States, over 16 million Spanish-speaking individuals are classified as having LEP [1]. Among Latino immigrants, those with LEP are less likely to receive psychiatric health care as compared to those with English proficiency (EP) [2,3]. Federal and state policies have been created to reduce language barriers to health care and mandate that interpreter services be available to all LEP individuals [4,5]. Human interpreters are considered the *gold standard* to provide linguistically and culturally competent health care to patients with LEP, leading to improvements in patient comprehension and satisfaction, clinical outcomes, and health care use [6]. However, the usage rate of these services remains low, as less than 20% of clinical encounters for patients with LEP use interpreting services, often due to time constraints for clinical encounters [7].

Currently, artificial intelligence (AI) interpretation technologies have already been implemented in a variety of industries as either a replacement for or augmentation to human interpretation [8]. Health care, however, has been slow to apply AI technologies. Moreover, there are limited published applications of AI interpretation in health care, despite promising early results for the use of AI interpretation for the translation of written text, including public health information and electronic health records [6,8,9]. Notably, a paucity of information exists on the application of AI interpretation in health care to *spoken* rather than written text.

Most publications regarding clinical interpretation focus on ways to optimize the experience of using an interpreter, and there are various guidelines that suggest strategies to best integrate the interpreter into the encounter [10]. It is frequently advised to use simplified speech, with pauses between sentences to allow for sentence-by-sentence translation. Some published simplifications include shortening of phrases as well as avoidance of complex language, including idiomatic expressions, jargon, and humor [10]. The extent to which patients condense and simplify their speech when using an interpreter is yet to be evaluated.

This paper describes the results of a cross-sectional study to evaluate the translational accuracy of a novel AI interpretation technological tool composed of dual automated speech recognition (ASR) and automated machine translation (AMT) function. *ATP App* was developed by the University of California, Davis team to transcribe and translate psychiatric interviews with Spanish-speaking patients who have LEP. When assessing translational accuracy, it is important to be aware that mistakes can occur at both the ASR transcription and the AMT translation stages of AI interpretation. A separate paper further describing the accuracy of the AI interpretation has been prepared (Chan S et al, unpublished data, 2021). This study focuses specifically on the ability of *ATP App* to translate complex, figurative language devices (FLDs) such as metaphors, similes, and euphemisms [11]. To maintain the original meaning of these devices, the technology must be capable of recognizing that a literal, word-for-word translation does not always confer semantic equivalence between a phrase in Spanish and English [12]. As such, the translation of FLDs is a complex task, but one that would be required of AI interpretation in its application to real-world patient-provider conversations.

This study also aimed to quantify the extent to which the use of an interpreter affects patient speech quantity, measured by patient word count per minute; it also aimed to understand whether patient speech quantity differed between in-person or videoconferencing environments, the latter being required during the COVID-19 pandemic [13]. As such, we hoped to objectively quantify some of the time and language content barriers that physicians and patients face when using interpreting services.

## Methods

### Ethics Approval

This study was nested within a larger clinical trial approved by the University of California, Davis Institutional Review Board (IRB reference number: 1131922; trial registration number: NCT0358860) [14].

### Participant Selection

The original study recruited Hispanic individuals with significant LEP from mental health and primary care clinics. All participants were aged 18 or older and screened as likely to have either a nonurgent psychiatric disorder, namely mood, anxiety or substance use disorders, or a chronic medical condition. Exclusion criteria included suicidal ideation or plans, significant cognitive deficits, and those otherwise deemed inappropriate for participation by their primary care provider or psychiatrist.

A total of 6 patients with psychiatric disorders were randomly selected from the original study of 114 patients. The first 3 (50%) patients were recruited prior to the COVID-19 pandemic, and the second 3 (50%) patients were recruited after the start of the pandemic. This allowed us to assess if the transition to a web-based, Zoom platform would impact AI interpretation.

### Interview Format

The participants underwent 2 methods of psychiatric assessments*.* Method A represented the current gold standard of interviews of patients with LEP, whereby the Spanish-speaking patient was interviewed by an English-speaking psychiatrist, and the interview was interpreted by a human, English-Spanish interpreter*.* This method is the language-discordant format, with the provider and patient speaking different languages. Method B represented the novel, asynchronous telepsychiatry (ATP), AI interpretation format whereby the Spanish-speaking patient was interviewed by a Spanish-speaking researcher-interviewer, who was trained to administer psychiatric interviews. These interviews were video and audio recorded and subsequently transcribed and translated into English with subtitles added to the video file. The files were then sent to an English-speaking psychiatrist for diagnosis and treatment plan recommendations. Asynchronous telepsychiatry, without the added component of language interpretation, has already been established as a clinically valid method for psychiatric assessments [15]. Transcription and translation were carried out via a novel, cloud-based, dual ASR and AMT app already developed by the research team, entitled *ATP App*. The videos were later viewed by the psychiatrist. This method is the language-concordant format, with the researcher-interviewer and patient speaking the same language. Of note, although it is common practice for human interpreters to *set the stage* and ask participants to simplify or shorten their speech to facilitate ease of interpretation, we specifically did not ask the participants to modify their speech in any way. This allowed us to analyze the natural speech of the encounters for both methods [9]. All interviews in both methods were video and audio recorded.

### Transcription and Translation

Transcripts for both methods were generated from the video/audio recording of each interview. These transcripts were initially generated automatically and were subsequently verified for accuracy and edited by 2 bilingual researchers. The verification process was a labor-intensive process, requiring each reviewer to replay the file multiple times to add, remove, and replace words. The process of transcript verification required approximately 4 minutes of editing per 1 minute of the interview (Chan S et al, unpublished data, 2021). Instances of use of FLDs spoken by the patient were then separately marked by 2 bilingual researchers. There is a wide variety of FLDs (eg, similes, metaphors, irony, idiomatic expressions, and euphemisms), all of which apply language in a nonliteral manner to add connotation [11]. Table 1 presents examples for some common types of FLDs. FLDs used by the interviewers were excluded from analysis to control for natural variation in the style of speech used by the interviewers.

**Table 1:** Example figurative language devices.

Figurative language device subtype	Example in Spanish	Correct translation into English	Literal translation into English
Metaphor	Eso se me está llenando el cerebro.	This is overwhelming me.	This is filling my brain.
Idiomatic expression	Me hacen bien pesado.	It’s been very hard.	They make me very heavy.
Simile	Me siento que no sirvo para nada.	I feel like I’m worthless.	I feel like I don’t serve for anything.
Personification	Se me despega mi cabeza.	I lose my mind.	I peel away my head.
Euphemism	Me sentía yo más decaída.	I felt more down.	I felt more droopy.
Hyperbole or exaggeration	No me muero de hambre.	I’m not going to starve to death.	I’m not going to die from hunger.

Accuracy of transcription and translation of each FLD was independently determined by 2 bilingual researchers. If an FLD was categorized as an *inaccurate transcription*, the FLD was marked as “transcript inaccurate,” and no subsequent analysis of translation was made, as translation is dependent on accurate transcription. If an FLD was categorized as an *accurate transcription*, the FLD was then subdivided into either an *accurate* or an *inaccurate translation*.

To analyze the quantity of patient speech, separate subtranscripts were created of only the patients’ speech to obtain a patient word count. This word count was then divided by the minutes of the interview, to control for varying lengths of interviews. The number of instances of FLDs was divided by the number of minutes of the interview to control for the varying lengths of patient interviews.

The primary statistical analysis compared FLD frequency per minute, patient word count per minute, and percentage of accurate translation of FLDs between Method A and Method B for each patient. Analysis was performed using Microsoft Excel with paired sample two-sided *t* tests. The secondary statistical analysis compared only the percentage of accurate translation of FLDs as stratified into the in-person, pre–COVID-19 group for patients 1-3, and the Zoom format, post–COVID-19 group for patients 4-6. *P*<.05 was used to determine significance for all analyses.

## Results

The study included 4 (67%) female and 2 (33%) male participants, with an age range of 42-71 years and an average age of 53 years; 4 (67%) participants were born in Mexico, 1 (17%) in Costa Rica, and 1 (17%) in Guatemala.

**Figure 1:**
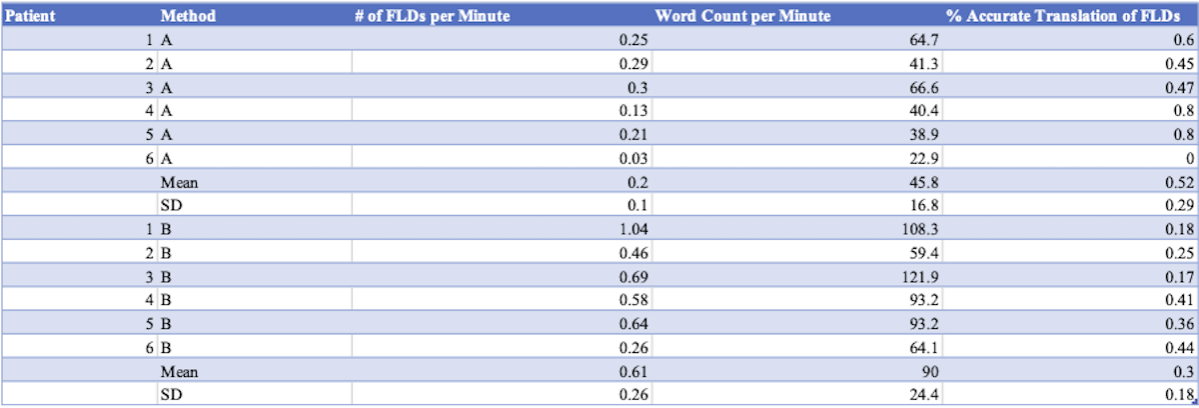
Frequency of figurative language devices, patient word count per minute, and percentage of accurate translation per method and patient. FLDs: figurative language devices.

Figure 1 details the results of the three primary comparisons between each method—the frequency of figurative language devices as measured by number of FLDs per minute, the patient word count per minute, and the percentage of accurate translation as measured by number of correctly translated FLDs per total number of FLDs. There was a significant increase in the per-minute frequency of FLDs using AI interpretation (mean 0.61, SD 0.26) compared to using the human interpreter: mean 0.2, SD 0.1; *t*_5_=–4.58, *P*≤.05. There was a significant increase in the per-minute patient word count using AI interpretation (mean 90, SD 24.4) as compared to using the human interpreter: mean 45.8, SD 16.8; *t*_5_=–7.7, *P*≤.05. There was an insignificant decrease in the mean percentage of accurate translation of FLDs using AI interpretation (mean 0.3, SD 0.18) compared to using the human interpreter: mean 0.52, SD 0.29; *t*_5_=1.59, *P*=.17.

Secondary comparisons were made to assess for possible differences in the percentage of accurate translation of FLDs for the interviews that were performed in person, prior to the COVID-19 pandemic, compared to those that were obtained over Zoom, after the COVID-19 pandemic. There was an insignificant increase in the accuracy of both methods for the Zoom format (mean 0.47, SD 0.3) as compared to the in-person format: mean 0.35, SD 0.17; *t*_5_=–0.95, *P*=.39. When broken down separately by method, however, there was a near significant increase in the accuracy of AI interpretation for the Zoom format (mean 0.4, SD 0.04) as compared to the in-person format: mean 0.2, SD 0.04; *t*_2_=–4.02, *P*=.06. There was an insignificant increase in the accuracy of human interpretation for the Zoom format (mean 0.53, SD 0.46) compared to the in-person format: mean 0.51, SD 0.08; *t*_2_=–0.1, *P*=.92.

## Discussion

### Principal Findings

This study looked at the linguistic differences in psychiatric interviews of Spanish-speaking patients with LEP. The results demonstrate that the patients’ speech differs significantly. Method A in the presence of a human interpreter showed fewer instances of FLDs, compared with Method B with language-concordant interviews augmented with AI interpretation. Additionally, in Method A, patients spoke with a lower word count per minute compared to Method B, with an average of half as many words per minute in the presence of a human interpreter. There was no statistically significant change in these results when using videoconferencing, compared to in-person consultations, although the interpreting accuracy over videoconferencing was higher for both methods.

Our findings aligned with our expectation that patient speech becomes simplified and truncated when using a human interpreter. This simplification aligns with many published guidelines and articles that detail best practices for use of human interpreting services, which often encourage a reduction in the use of idiomatic speech, as well as a simplification of sentence structure [10]. Within the specialty of psychiatry, diagnosis and treatment decisions are heavily reliant on the verbal history conveyed to the provider [16]. Our results suggest that the history provided using a human interpreter will likely differ and could represent a less comprehensive picture of the patient’s psychopathology. Of note, human interpreting services guidelines are generally geared toward providers rather than patients, and the patients included in our study would likely not have read these guidelines prior to the study. Instead, we propose that there is an innate tendency for the patients to simplify their speech when having to pause between sentences to allow for translation. Additionally, the use of a human interpreter has previously been associated with a reduced number of follow-up appointments, reduced patient and provider satisfaction, and an increased likelihood of not asking the questions that the patient wanted to ask [17-19].

Moreover, the results of our study demonstrate that the use of an in-person human interpreter (Method A) is currently more accurate than AI interpretation (Method B) regarding the translation of FLDs. The aggregate translational accuracy for human interpreters was 52% versus 30% for AI interpretation (*P*>.05), suggesting that both methods lend themselves to a high degree of inaccuracy when translating FLDs. Of note, a sizable contribution to the inaccuracy of translation by the AMT starts from an inaccurate transcription of the conversation, suggesting that improvements in audio recording and transcription would increase the translational accuracy of the AI interpretation.

Finally, our results show that the transition of interviews from in-person to the web-based, Zoom format in response to the COVID-19 pandemic led to a higher, but statistically insignificant percentage of translational accuracy of FLDs, suggesting that both human-interpretation and AI interpretation technologies can be adapted to accommodate the movement away from in-person psychiatric evaluations. The aggregate translational accuracy of Method A is 50% in-person vs 53% over Zoom, and the aggregate translational accuracy of Method B is 20% in-person vs 40% over Zoom. This difference appears to stem from an improvement in *transcriptional* accuracy on the Zoom format, likely seen because interview participants took longer pauses after speaking and spoke in shorter phrases over the Zoom format.

### Limitations

There are several limitations that we have identified in this study. First, the study is limited due to the small panel of patient interviews that are included. The decision to analyze a limited subset of 12 patient interviews from the initial cohort of approximately 200 patient interviews was made due to the significant time required to both generate transcriptions for the in-person Method A and to verify the machine-generated transcripts for accuracy for Method B. Expanding the sample size of the included patient interviews is possible in the future using our database of recorded interviews but will be time consuming. This study is additionally limited by the wide variety of types of FLDs used in the interview discourse. Some devices, such as idioms and metaphors, are clear to delineate from nonfigurative speech. For example, the following patient statement, “estoy viendo una luz al final del túnel” (“I am seeing a light at the end of the tunnel”) is clear to recognize as a figurative language device; it is well understood that the patient is not actually seeing a light, but rather that they are using an idiom that is in common use in both the English and the Spanish languages. By contrast, some of the types of devices that are used less frequently (eg, personification and euphemism) are more subtle. For example, the following patient statement, “la enfermedad me hizo traermelo para acá” (or “the sickness made me bring him too”) is less obvious to recognize as figurative language, whereby her depression (“the sickness”) is personified to have forced the patient to do something.

### Highlights

- Patients with LEP frequently receive substandard health care because of language communication difficulties. Medical interpreters are often in short supply and commonly lengthen the time and simplify the language of medical interviews.
- A combination of ASR and AMT technologies have been developed as a method of AI interpretation. We applied these to ATP consultations as we believe AI interpretation may be a way of improving psychiatric interviews across languages compared with interviews mediated through human interpretation.
- In this study, the number of FLDs, the translation accuracy of figurative language, and the patient word counts were compared as proxies for interview complexity and volume. We found in the AI interpretation model that word counts were greater, and FLDs were more common but less accurately translated than in the human interpreter model.

### Conclusion

Going forward, technological improvements of AI interpretation from both the transcription component and the translation component will be required for ATP interviews to be conducted in languages other than English. The field of AI interpretation has made substantial progress within the past decade with the transition from statistical machine translation to neural machine translation [20]; we expect that AI interpretation will continue to expand and improve in the coming years and to eventually be at least as accurate as professional interpreters, allowing it to be introduced into regular clinical use. As our patient population in the United States continues to diversify, it will be important to further develop novel technological approaches to circumvent the time limitations and simplification of speech that are currently seen with human interpretation. Further studies of the accuracy of interpretation over videoconferencing compared with in-person interpreting are required.

## Abbreviations

AI: artificial intelligence
AMT: automated machine translation
ASR: automated speech recognition
ATP: asynchronous telepsychiatry
FLD: figurative language device
LEP: limited English proficiency
